# Motivational Interviewing in a Web-Based Physical Activity Intervention With an Avatar: Randomized Controlled Trial

**DOI:** 10.2196/jmir.2974

**Published:** 2014-02-13

**Authors:** Stijn Friederichs, Catherine Bolman, Anke Oenema, Janneke Guyaux, Lilian Lechner

**Affiliations:** ^1^Faculty of Psychology and Educational SciencesOpen University of the NetherlandsHeerlenNetherlands; ^2^Department of Health PromotionMaastricht UniversityMaastrichtNetherlands

**Keywords:** motivational interviewing, physical activity, Internet, avatar

## Abstract

**Background:**

Developing Web-based physical activity (PA) interventions based on motivational interviewing (MI) could increase the availability and reach of MI techniques for PA promotion. Integrating an avatar in such an intervention could lead to more positive appreciation and higher efficacy of the intervention, compared to an intervention that is purely text-based.

**Objective:**

The present study aims to determine whether a Web-based PA intervention based on MI with an avatar results in more positive appreciation and higher effectiveness of the intervention, when compared to an intervention that is purely text-based.

**Methods:**

A three-arm randomized controlled trial was conducted, containing the following research conditions: (1) a Web-based PA intervention based on MI with an avatar, (2) a content-identical intervention without an avatar, and (3) a control condition that received no intervention. Measurements included PA behavior and process variables, measured at baseline, directly following the intervention and 1 month post intervention.

**Results:**

Both interventions significantly increased self-reported PA at 1 month, compared to the control condition (beta_AVATARvsCONTROL_=.39, *P*=.011; beta_TEXTvsCONTROL_=.44, *P*=.006). No distinctions were found regarding intervention effect on PA between both interventions. Similarly, the results of the process evaluation did not indicate any significant differences between both interventions. Due to the limited relational skills of the avatar in this study, it probably did not succeed in forming a stronger relationship with the user, over and above text alone.

**Conclusions:**

The findings suggest that avatars that do not strengthen the social relationship with the user do not enhance the intervention impact. Future research should determine whether Web-based PA interventions based on MI could benefit from inclusion of a virtual coach capable of more complex relational skills than used in the current study, such as responding in gesture to the user’s state and input.

**Trial Registration:**

Dutch Trial Register trial number: NTR3147; http://www.trialregister.nl/trialreg/admin/rctview.asp?TC=3147 (Archived by WebCite at http://www.webcitation.org/6NCbwdUJX).

## Introduction

Motivational interviewing (MI) is defined as “a collaborative conversation style for strengthening a person’s own motivation and commitment to change” [1]. Evidence illustrates that MI can be successful in getting individuals to increase their physical activity (PA) [2]. Delivering MI in a traditional way, however, is expensive and therefore difficult to scale up. Therefore, developing Web-based PA interventions based on MI could increase the availability and reach of the MI techniques for PA promotion.

Although developing a Web-based PA intervention based on MI appears feasible [3], questions remain to be answered regarding the optimal configuration of such an intervention. MI is usually delivered as a face-to-face intervention by a counselor. In the context of a Web-based delivery mode, the presence of the human counselor could be substituted by using a virtual agent or an avatar [4]. Multiple studies have confirmed that the presence of a virtual agent can further improve effectiveness of Web-based interventions [4,5].

Considering the above, integrating an avatar into a Web-based PA intervention based on MI could lead to more positive appreciation and higher efficacy of the MI components. Future intervention development would be less expensive, however, when such an avatar is omitted. Thus, it is important to determine whether the addition of an avatar leads to more favorable results in the context of a Web-based PA intervention based on MI.

The present study aims to answer the following questions: (1) Does adding an avatar to a Web-based PA intervention based on MI result in additional effects on PA behavior? and (2) Does the presence of such an avatar lead to better appreciation for the intervention?

## Methods

### Overview

A three-arm randomized controlled trial was conducted, containing the following research conditions: (1) a Web-based PA intervention based on MI with an avatar (AVATAR), (2) a content-identical intervention without an avatar (TEXT), and (3) a control condition that received no intervention (CONTROL). Measurements were taken using Web-based questionnaires at baseline, directly after the intervention (follow-up 1) and 1 month post intervention (follow-up 2).

### Participants

The participants were Dutch adults (18-70 years old). Exclusion criteria included impairments that severely impede PA participation (participants were asked whether they were unable to be physically active), not speaking and/or writing Dutch, and not having Internet access. The participants were recruited in April and May 2012 through an Internet panel of Dutch residents who occasionally volunteer in Web-based research.

### Measurements

At baseline and follow-up 2, the number of weekly days with at least 30 minutes of moderate PA was measured with a self-reported single item of the Dutch Short Questionnaire to Assess Health Enhancing Physical Activity (SQUASH): “How many days per week are you, in total, moderately physically active, by undertaking, for example, heavy walking, cycling, chores, gardening, sports, or other physical activities for at least 30 minutes?” [6]. At follow-up 1, appreciation for the intervention was evaluated by measuring several appreciation dimensions such as personal relevance, trustworthiness, and overall appreciation.

### Interventions

Two Web-based PA interventions based on MI were developed, one of which included an avatar (AVATAR) and one of which was fully text-based (TEXT). Both interventions were derived from a previous study on a Web-based PA intervention based on MI [3]. In these interventions, participants answered several open-ended and multiple choice questions. In between those questions, at various instances, the participants received feedback messages containing a reflection or summary, based on one or more of their prior answers. The following is an example of such a message:

So you have a very busy life, and therefore you have less confidence that you could manage to increase your physical activity, which is completely understandable. However, you also said that you’re a real go-getter. When you have decided to do something, you go all the way! Because of that, you still have confidence that you could increase your physical activity. Do you already have some ideas about how you would increase your physical activity? What activity would you want to do? And how could you schedule this in such a way that it would not take too much time? With your willpower and a good plan, you would surely be able to become more physically active.

During both interventions, several topics are discussed such as the participant’s current PA behavior, the perceived importance of PA and potential beneficial effects of becoming more physically active, and the participant’s confidence that he or she could succeed in becoming more physically active. Finally, participants are given the option to formulate their own PA plans and to anticipate difficulties.

Throughout both interventions, several MI counseling techniques have been implemented and have been translated into automatized text versions. Obviously, application of these techniques by means of an automated Web-based platform differs from application in a real-life counseling setting. For example, a face-to-face setting allows responding to very subtle expressions of motivation, which is less feasible in an automated platform. Due to the specific and interactive approach, however, the application of these techniques in the interventions displays a high degree of similarity to the real-life situation, in which a counselor asks questions and provides feedback. The main difference is that the questions and feedback are not provided by a human counselor, but through text or an avatar on a computer screen.

With regard to textual content, the TEXT and AVATAR interventions are almost completely identical. Throughout both interventions, the feedback messages are identical. During a few moments in the intervention, however, the AVATAR intervention contains somewhat more social dialogue than the TEXT intervention.

**Figure 1:**
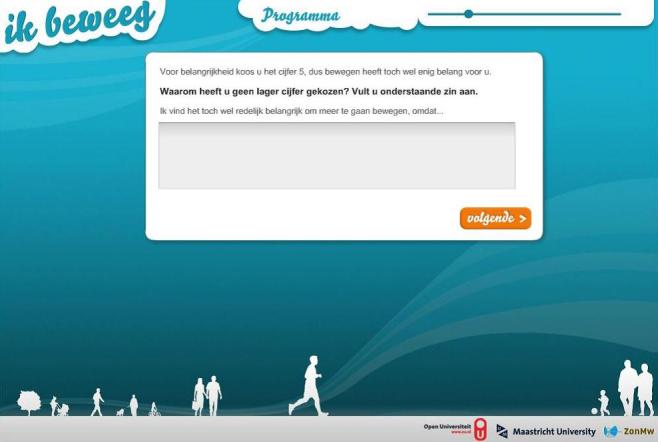
Screenshot of the TEXT intervention.

The TEXT intervention has a relatively simple layout, consisting of a static blue background and a white dialogue window where questions and feedback messages appear. Figure 1 shows a screenshot of the TEXT intervention.

**Figure 2:**
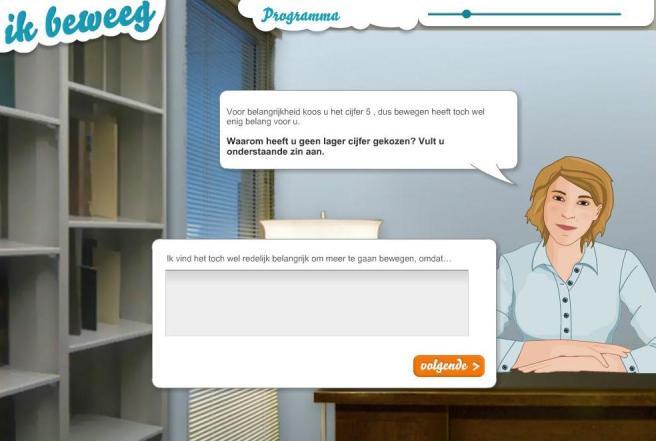
Screenshot of the AVATAR intervention.

The AVATAR intervention consists of an avatar positioned behind a desk in a small office. Questions and messages are communicated through text balloons, without the use of voice. The avatar displays speech movements (matching the text in the balloons), social dialogue (at the beginning of the intervention and during transitions between the different parts of the intervention) as well as non-verbal expressions such as empathic gestures and eye and head movements. The physical appearance of the avatar was based on the results of a series of focus group interviews among the target population that discussed how a motivating and reliable avatar should look. Based on the results of these interviews, it was decided to include both a male and a female avatar in the intervention so the participants can choose a coach in accordance with their preferences. At the start of the intervention, participants can choose either a male or a female avatar. Figure 2 shows a screenshot of the AVATAR intervention. Before implementation, the intervention was extensively pretested by members of the target group.

### Statistical Analyses

Physical activity data were analyzed using a linear regression analysis with the following independent variables: baseline PA behavior, gender, age, body mass index (BMI), educational level, and the intervention condition variable coded into two dummies (AVATAR and TEXT). Potential variations in process evaluation variables were analyzed through analysis of covariance (ANCOVA) with gender, age, BMI, educational level, and baseline PA behavior as covariates. Analyses were performed using SPSS for Windows (version 18).

## Results

At baseline, 958 individuals (60.4% female, 578/958; mean age 42.9 [SD 14.5]; 58% high education, 555/958) completed the questionnaire. These individuals were on average moderately physically active for at least 30 minutes per day an average of 4.2 (SD 1.9) days per week. At baseline, the TEXT condition contained significantly fewer participants with high education compared to the AVATAR and CONTROL condition. No additional baseline differences were found between the three study conditions.

Follow-up 2 (1 month) measurements were completed by 500 participants (AVATAR 162; TEXT 146; CONTROL 192) or 52% of the baseline population (57.8% female, 289/500; mean age 45.3 [SD 14.2]; 58% high education, 290/500). Dropout analyses showed that participants younger than age 46 were more likely to drop out at 1 month (OR 1.95, 95% CI 1.51-2.53). In addition, participants randomized into one of the intervention conditions were more likely to drop out at 1 month (OR 1.64, 95% CI 1.25-2.16).

At 1 month, participants were on average moderately physically active for an average of 4.4 (SD 1.8) days per week for at least 30 minutes per day (AVATAR mean 4.6 [SD 1.6]; TEXT mean 4.7 [SD 1.8]; CONTROL mean 4.0 [SD 1.9]). Both interventions were effective in increasing total PA at 1 month when compared to the control condition (beta_AVATARvsCONTROL_=.39, *P*=.011; beta_TEXTvsCONTROL_=.44, *P*=.006). Participants from the two intervention conditions who had completed the follow-up 2 questionnaire (n=308) increased the number of days per week on which they were physically active for at least 30 minutes from 4.44 to 4.63 (AVATAR from 4.43 to 4.57; TEXT from 4.46 to 4.69). No differences were found regarding intervention effect on PA level between AVATAR and TEXT condition.

Overall, process evaluation results were quite positive (ie, entertainment 5.16/7; trustworthiness 5.15/7; overall appreciation score 7.14/10). No significant differences were found between the intervention conditions regarding these variables.

## Discussion

### Principal Findings

Both interventions significantly increased self-reported PA at 1 month, compared to the control condition. These outcomes indicate that Web-based PA interventions based on MI hold promise, as they are potentially capable of inducing behavior change. No distinctions were found regarding effect on PA level between the AVATAR and TEXT intervention. Similarly, the results of the process evaluation did not indicate any significant differences between both interventions.

The avatar in this study did not increase intervention impact. This is probably related to the inability of the avatar to respond in gestures to the user’s state and input [5]. Due to the limited relational skills of the avatar in this study, it probably did not succeed in forming a stronger relationship with the user, over and above text alone. As a consequence, the avatar used in the current study was not able to enhance the intervention. For future intervention research, inclusion of an avatar capable of more sophisticated relational skills, such as responding to the user’s input with gestures, is warranted.

Alternatively, it would be relevant to test a modality in which the avatar is replaced by multiple short videos of a real human who speaks to the participant and leads the way through the intervention. Previous research shows that this type of interactive video counseling holds promise for public health interventions [7]. These videos could even be supplemented by videos in which a PA expert talks about the possible benefits of being physically active on a regular basis, or in which former participants talk about their experiences during the intervention. All this could help give the Web-based intervention a more human character.

### Limitations

This study has some limitations. First, a considerable degree of self-selection occurred in this study, due to the fact that participants from the intervention conditions were more likely to drop out compared to participants from the control conditions. This may be related to a second consideration; participating in this study was relatively demanding since the baseline measurement, intervention, and post-measurement were all on the same day. This may have led to some irritation among the participants and probably explains a part of the relatively large attrition that occurred in the intervention conditions of this study. Third, PA behavior was measured with a single self-report item. Although several studies have provided support for the validity of the item used in this study [8,9], the measure remains a weakness of the current study and an objective measure of PA (eg, by pedometer or accelerometer) is recommended for future studies. Finally, caution is needed when generalizing the results of this study to the general population because of the overrepresentation of a high educational level in the sample due to the Web-based sampling frame [10].

### Conclusion

In conclusion, the findings suggest that avatars that do not strengthen the social relationship with the user do not enhance intervention impact. Future research should determine whether Web-based PA interventions based on MI could benefit from inclusion of a virtual coach capable of more complex relational skills than used in the current study, such as responding with gestures to the user’s state and input [5]. Furthermore, future research should assess the use of video coaching as a potentially beneficial part of a Web-based PA intervention based on MI.

## Abbreviations

ANCOVA: analysis of covariance
BMI: body mass index
MI: motivational interviewing
OR: odds ratio
PA: physical activity
SQUASH: Short Questionnaire to Assess Health-Enhancing Physical Activity
